# Diagnosis of diffuse idiopathic skeletal hyperostosis with chest computed tomography: inter-observer agreement

**DOI:** 10.1007/s00330-016-4355-x

**Published:** 2016-04-20

**Authors:** S. F. Oudkerk, Pim A. de Jong, M. Attrach, T. Luijkx, C. F. Buckens, W. P. Th. M. Mali, F. C. Oner, D. L. Resnick, R. Vliegenthart, J. J. Verlaan

**Affiliations:** 1 Department of Radiology and Nuclear Medicine, University Medical Center Utrecht, Room E01.132, 3508 GA Utrecht, Netherlands; 2 Department of Orthopedics, University Medical Center Utrecht, Utrecht, Netherlands; 3 Division of Musculoskeletal Radiology, Department of Radiology, University of California, San Diego School of Medicine, San Diego, CA USA; 4 Center for Medical Imaging – North East Netherlands, University of Groningen, University Medical Center Groningen, Groningen, The Netherlands; 5 Department of Radiology, University of Groningen, University Medical Center Groningen, Groningen, The Netherlands

**Keywords:** Diffuse idiopathic skeletal hyperostosis (DISH), CT, Thorax, Inter observer agreement, Resnick criteria

## Abstract

**Objective:**

To evaluate and improve the interobserver agreement for the CT-based diagnosis of diffuse idiopathic skeletal hyperostosis (DISH).

**Methods:**

Six hundred participants of the CT arm of a lung cancer screening trial were randomly divided into two groups. The first 300 CTs were scored by five observers for the presence of DISH based on the original Resnick criteria for radiographs. After analysis of the data a consensus meeting was organised and the criteria were slightly modified regarding the definition of ‘contiguous’, the definition of ‘flowing ossifications’ and the viewing plane and window level. Subsequently, the second set of 300 CTs was scored by the same observers. κ ≥ 0.61 was considered good agreement.

**Results:**

The 600 male participants were on average 63.5 (SD 5.3) years old and had smoked on average 38.0 pack-years. In the first round κ values ranged from 0.32 to 0.74 and 7 out of 10 values were below 0.61. After the consensus meeting the interobserver agreement ranged from 0.51 to 0.86 and 3 out of 10 values were below 0.61. The agreement improved significantly.

**Conclusions:**

This is the first study that reports interobserver agreement for the diagnosis of DISH on chest CT, showing mostly good agreement for modified Resnick criteria.

**Key Points:**

• *DISH is diagnosed on fluoroscopic and radiographic examinations using Resnick criteria*

• *Evaluation of DISH on chest CT was modestly reproducible with the Resnick criteria*

• *A consensus meeting and Resnick criteria modification improved inter-rater reliability for DISH*

• *Reproducible CT criteria for DISH aids research into this poorly understood entity*

## Introduction

Diffuse idiopathic skeletal hyperostosis (DISH) is a disorder involving ossification of ligaments and bone proliferation at entheses. Characteristically (and by definition), it affects the thoracic spine [1]. DISH is a condition of the elderly and is rarely seen before middle age. It is more common in men than in women; ratios between men/women vary between 2:1 and 7:1 [2, 3]. The ossification, suggested to originate from the spinal longitudinal ligaments (especially from the anterior longitudinal ligament), produces a cascading pattern of paravertebral bone formation, especially along the anterolateral aspect of the vertebral bodies [4].

The most commonly used diagnostic criteria were established by Resnick and Niwayama in 1976 and required involvement of at least four contiguous vertebrae of the thoracic spine, preservation of the intervertebral disc space, and absence of gross degeneration or fusion of the apophyseal and sacroiliac joints [5]. The criterion of four contiguous vertebrae with flowing bridging ossifications enabled standardization of the diagnosis but did not lead to consensus about the number of levels needed to be involved. Several other sets of classification criteria have been proposed in the past with different numbers of connecting vertebrae [6, 7]. The Resnick criteria on preservation of the intervertebral disc space and absence of inflammatory/degenerative changes in the apophyseal and sacroiliac joints are useful to exclude previous spondylodiscitis, disc degeneration and ankylosing spondylitis as alternative causes for bridging ossifications.

The underlying pathogenetic mechanism of DISH is poorly understood, but genetic, metabolic, endocrinologic, anatomic, environmental and toxic factors possibly contribute to the development of DISH [8–11]. Specifically, the association of the metabolic syndrome and development of DISH has been shown by various authors [12]. The criteria Resnick established in 1976 stem from an era when computed tomography (CT) had yet to develop as a diagnostic tool. CT provides far more detailed evaluation of the intervertebral disc spaces and bridging ossifications, but the observer agreement of a CT-based diagnosis of DISH is unknown.

There is growing awareness that the presence of DISH is associated with morbidity and mortality, especially in the setting of trauma and cardiovascular events [13–15]. Currently, DISH is usually observed as an incidental finding on imaging performed for other reasons [14]. Chest CT is frequently requested and allows detailed visualization of the thoracic spine. The reproducibility of chest CT-based diagnosis of DISH is therefore of interest for clinical care and may facilitate research into the aetiology of DISH.

The purpose of this study was to evaluate the interobserver agreement for the CT-based diagnosis of DISH.

## Material and methods

### Study population

This is a side study of the Dutch Belgian Lung Cancer Screening Trial (NELSON-trial ISRCTN63545820) [16]. The trial was approved by the Dutch Ministry of Health and by the institutional ethics review board of the participating hospitals. Informed consent was obtained from all study participants. The trial included current and former smokers between the ages of 50 and 75 years with at least 16.5 pack-years of smoking history who were physically fit enough to potentially undergo surgery. For this study, we included a sample of 600 male participants from the University Medical Center Utrecht. The sample was randomly divided into two groups of 300 male participants. Detailed characteristics of the sample are given in Table 1 (group I and II).

**Table 1:** Subject characteristics of the sample (group I and II combined)

Characteristics	Value
Age (years), mean ± SD	63.5 ± 5.3
BMI (kg/m^2^), mean ± SD	27.5 ± 5.3
Smoking status, *n* (%)	
Current smoker	302 (50.3)
Former smoker	298 (49.7)
Pack-years (years), median (25th–75th percentile)	38.0 (29.7–49.5)

NB: All subjects were male

### CT

Volumetric CT in inspiration was obtained in the craniocaudal direction after standardized breathing instructions by a trained radiographer. CT images were acquired with 16 × 0.75 mm collimation (Brilliance 16P; Philips Medical Systems, USA), and images with slice thickness of 1.0 mm at 0.7-mm increment were reconstructed using a smooth kernel (B-filter; Philips). Dose settings were adjusted to body weight: subjects weighing 80 kg or less received 120 kVp at 30 mAs and subjects weighing over 80 kg received 140 kVp at 30 mAs.

### Visual evaluation of CT images

Five independent observers with various levels of expertise in evaluating chest CT images and differing in background (radiology or orthopaedic surgery) participated in this study: one radiologist who specialized in chest radiology, one orthopaedic surgeon with expertise in DISH, one senior resident in radiology with a chest radiology specialty, and two junior residents in radiology (Table 2) [17]. A musculoskeletal radiologist responsible for the original set of criteria for establishing the diagnosis DISH aided in the study design and modification of the criteria. All CT images were presented to the observers in a randomized order on a 3D research workstation (iXviewer, Image Sciences Institute, Utrecht, the Netherlands). The observers were able to view each scan in any plane desired corresponding to regular practice. The CT window level for each scan was initially set at W/L 800/2000; this was a standard bone window setting that could subsequently be altered by the observer.

**Table 2:** Observers skills and level of expertise for diagnosing DISH on chest CT

	Job title	Expertise level^a^	Reading chest CT^b^
Observer 1	Junior resident	II	3
Observer 2	Junior resident	II	3
Observer 3	Senior resident	III	5
Observer 4	Orthopaedic surgeon	V	10
Observer 5	Chest radiologist	V	15

^a^Level of expertise based on ref. [8]: I = has knowledge and some skills; II = acts under strict supervision; III = acts under limited supervision; IV = acts without supervision; V = supervises and teaches

^b^Years since the observer started reading and evaluating chest CT scans

In the first round, the observers were asked to judge the presence or absence of DISH based on the Resnick criteria for the thoracic spine. The criteria were “(a) the presence of “flowing” calcification and ossification along the anterolateral aspects of at least four contiguous vertebral bodies with or without associated localized pointed bony excrescences at the intervening vertebral body–disc junctions; (b) a relative preservation of disc height in the involved areas and the absence of extensive radiographic changes of “degenerative” disc disease, including vacuum phenomena and vertebral body marginal sclerosis; (c) absence of apophyseal joint bony ankylosis” [5]. The fourth criterion of ‘absence of fusion of sacroiliac joints’ to differentiate DISH from ankylosing spondylitis was not used since the pelvic area was not available for review on the chest CTs used. It is suggested, however, that ankylosing spondylitis and DISH can be sufficiently distinguished on CT data.

**Fig. 1:**
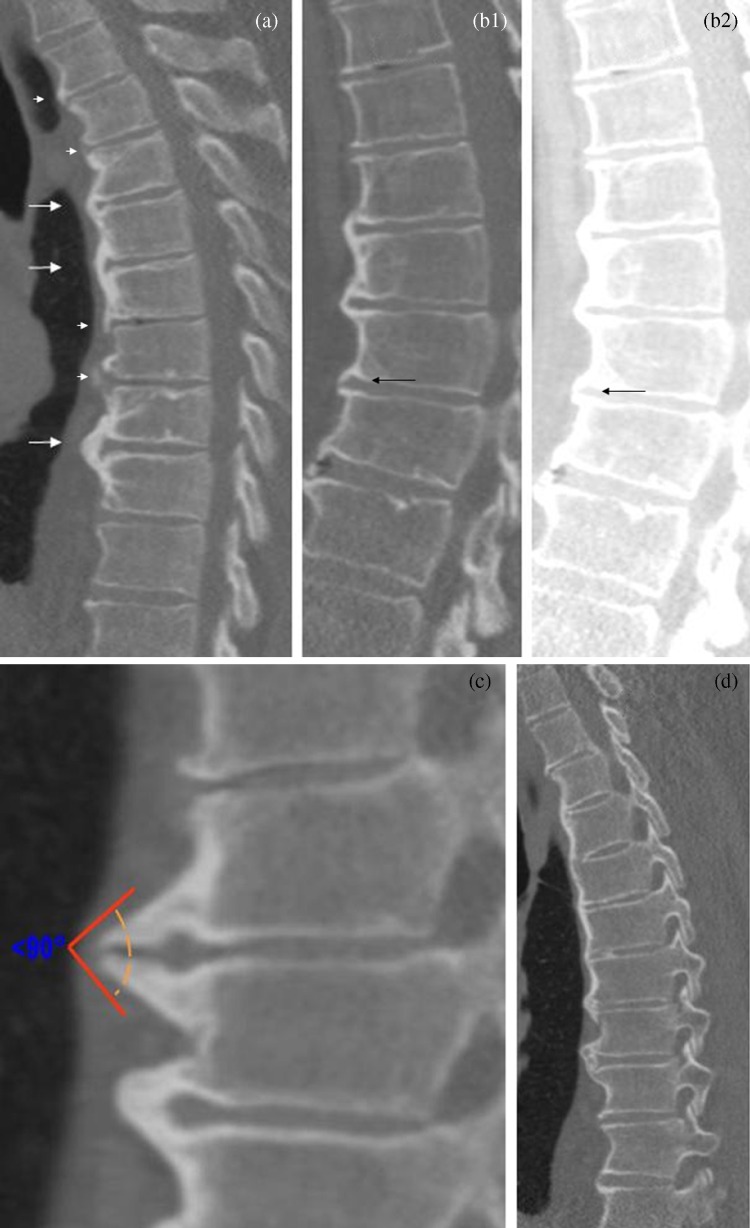
DISH criteria clarifications. **a** Shows 3 levels of DISH discontinuous flowing ossifications (*white arrows*); 3 contiguous interdisc levels are needed. The non-flowing or partial ossifications are marked with *arrowheads*. **b1**, **b2** By changing the W/L a spine can simulate DISH or flowing osteophyte (*arrows*). **c** Difference between degenerative osteophytes and DISH; less than 90° and sharp corners indicate degenerative disease. The vertebrae level below shows a flowing osteophyte with less than 90° corner but no sharp corners. **d** The pattern of flowing ossifications is typical of DISH and not characteristic of osteoarthritic degenerative disease. There is no facet ankylosis and mild discopathy; flowing ossifications are leading

Each case was evaluated in a first round by all observers independently without a consensus meeting. Before the second round κ values of the first round were calculated and presented, and ten cases scored differently by the observers were discussed in a consensus meeting which raised the following points. Firstly, regarding the definition of four contiguous vertebra, some observers used four intervertebral levels (i.e. four discs; five vertebral bodies) to define DISH, while others used four vertebrae and thus three intervertebral levels. Secondly, regarding the definition of flowing bridging ossifications, some observers scored any bridging ossification, while others, in view of the requirement for the ossifications to be ‘flowing’, used more strict criteria including a global angle of the bony bridge of more than 90° of the bridge or a bridge of similar thickness along its length and not substantially thicker at the vertebral level compared to the disc level. Thirdly, the presence of intervertebral disc degeneration as exclusion criterion for DISH was discussed. While some observers permitted spines with mild degeneration of the disc to still qualify for a diagnosis of DISH, others did not. Finally, the impression that altering window setting and viewing directions might introduce substantial bias was expressed by all observers. On the basis of the ensuing discussion, a set of reference images was defined and the DISH criteria were refined with four clarifications (Fig. 1):

I. DISH is established when (at least) four contiguous vertebrae or, alternatively, three contiguous disc levels are bridged (Fig. 1a).
II. Window width and level require fixed (Bone) settings (Fig. 1b1, b2) to prevent false positive and false negative cases, which may result from changing the density of longitudinal ligaments. It was also decided to use a single viewing plane to limit observer variation and we uniformly chose the sagittal plane to optimally assess DISH.
III. The angle formed by an osteophyte in relation to vertebral bodies should be larger than 90° to differentiate flowing ossification from bridging degenerative osteophytes (Fig. 1c).
IV. All agreed that flowing ossifications are a hallmark of DISH and subsequently it was suggested to put less weight on disc changes as exclusion criterion. As a result, in cases of mild or moderate degenerative disc changes in combination with flowing ossifications the diagnosis DISH could be established. In cases of severe degenerative (disc) changes the diagnosis should not be established (Fig. 1d).

Subsequently, a second set of 300 CT scans were scored by the same five observers using the modified criteria:

I. The scan is viewed exclusively in the sagittal viewing plane for the purpose of diagnosing DISH.
II. The scan is viewed in a fixed window level of W/L 800/2000.
III. The outer contour of the flowing ossifications intersects the vertebral body at >90° respecting the globally flowing character of the bridging ossification.
IV. Severe disc degeneration excludes the diagnosis of DISH.
V. A minimum of three contiguous intervertebral levels or four contiguous vertebrae is needed with connecting flowing ossifications.

### Statistical analysis

Kappa (κ) values were calculated to assess interobserver agreement. Agreement was classified as poor when κ was 0.20 or less; fair when between 0.21 and 0.40; moderate when between 0.41 and 0.60; good when between 0.61 and 0.80; and excellent when higher than 0.80 [11]. All analyses were performed with SPSS version 15.0 for Windows (SPSS, Chicago, Illinois, USA).

## Results

### Study population

In accordance with the NELSON study population, smoking history was substantial. Approximately half of the patients were current smokers and the average age was slightly above 60 years (Table 1). Patients from group 1 (*n* = 289) and group 2 (*n* = 296) were used for κ calculation after the exclusion of 11 and four cases, respectively, for technical reasons. The prevalence of DISH when averaging the results of the five observers was 26 % and 21 % for group I and II, respectively.

### Observer agreement of CT-based evaluation of DISH group I

The interobserver agreement ranged from a κ value of 0.32 to 0.74 (median 0.57) i.e. between fair and good. A good κ > 0.61 was achieved for three out of 10 comparisons (Table 3).

**Table 3:** Interobserver agreement for the diagnosis DISH in group I before the consensus meeting

	Observer 1	Observer 2	Observer 3	Observer 4	Observer 5
Observer 1					
Observer 2	0.72 [0.63–0.80]				
Observer 3	0.41 [0.30–0.51]	0.32 [0.23–0.40]			
Observer 4	0.44 [0.34–0.53]	0.33 [0.24–0.42]	0.74 [0.62–0.85]		
Observer 5	0.67 [0.59–0.77]	0.60 [0.51–0.69]	0.57 [0.44–0.67]	0.57 [0.44–0.67]	

Data given are kappa (κ) values with confidence intervals (CI) in brackets

### Observer agreement CT-based evaluation of DISH group II

The κ values of interobserver agreement for the second group, scored after the consensus meeting, increased and ranged from a κ value of 0.51 to 0.86 (median 0.67) i.e. between moderate and excellent. Furthermore, a good or excellent κ > 0.61 was obtained for seven of the 10 comparisons (Table 4).

**Table 4:** Interobserver agreement group II

	Observer 1	Observer 2	Observer 3	Observer 4	Observer 5
Observer 1					
Observer 2	0.66 [0.55–0.76]				
Observer 3	0.62 [0.51–0.72]	0.86 [0.77–0.93]			
Observer 4	0.68 [0.57–0.78]	0.83 [0.74–0.91]	0.81 [0.71–0.90]		
Observer 5	0.81 [0.74–0.88]	0.57 [0.46–0.67]	0.51 [0.41–0.63]	0.59 [0.48–0.68]	

Data given are kappa (κ) values with confidence intervals (CI) in brackets

To compare the κ values a Fleiss’ kappa with a bootstrap confidence interval was calculated for group I and II. The values were 0.52 (95 % CI 0.45–0.59) and 0.68 (95 % CI 0.60–0.74), respectively, showing a significant improvement in observer agreement.

## Discussion

This is the first study evaluating interobserver agreement related to the diagnosis of DISH on chest CTs. Without a consensus meeting and with the current Resnick criteria, κ values ranging between 0.32 and 0.74 were found. Values lower than 0.40 are usually considered indicative of fair or poor interobserver agreement, suggesting that the reliability of the original Resnick criteria on chest CT may be problematic [18]. Modifications to the original definition by Resnick and Niwayama, developed during the consensus meeting, reduced the ambiguity of the criteria on chest CT amongst the observers. These modifications, along with a fixed viewing plane and window setting, improved the reproducibility significantly. This indicates that the modified Resnick criteria can be useful in daily practice to diagnose DISH.

The “strict radiological features” described by Resnick in 1976 were intended to be applied to conventional two-dimensional radiographs and therefore predate the widespread use of three-dimensional CTs [5]. Low dose CT (<1 mSv) of the spine has already shown superior image quality in terms of anatomical and diagnostic information [19]. CT allows for much more detailed evaluation of paravertebral ossifications and degenerative changes, which supports the conclusion that modifications of the Resnick criteria are necessary when applied to CT directly. The modifications we propose in this study may allow a more accurate diagnosis of DISH based on CT.

Our study may be of relevance to further elucidate the causes and consequences of DISH, which are currently largely unknown. Prior anecdotal observations and case reports describe pulmonary restriction in cases of DISH [20]. Also the association of the metabolic syndrome and development of DISH has been previously suggested [12]. The cause of DISH is probably multifactorial and some evidence points to an underlying systemic low-grade inflammatory process [8–11]. We acknowledge that modifying the rather arbitrary original criteria does nothing for the clarification of pathogenesis or aetiology of DISH. Nevertheless a reproducible method to establish the diagnosis is urgently needed for further aetiological research.

A strength of this study was the use of multiple observers with different levels of experience from multiple medical disciplines and a sufficient number of cases with DISH. The only previous study that tested observer agreement for the diagnosis of DISH was published in 1998 and used routine chest radiographs rather than CT [21]. That study included 55 patients with DISH and assessed the inter-rater reliability with the alpha statistic (0.44 to 0.71) for the thoracic spine.

The main limitation of this study is that the effect of the consensus meeting cannot be separated from the effect of modifying the Resnick criteria. The improved observer agreement may thus be an effect of the consensus meeting, the modified criteria or both. Nevertheless, it is suggested that our proposed modifications are important to achieve good agreement between observers when diagnosing DISH on CT. A second limitation is the definition of flowing ossification. We decided to strictly adhere to a sagittal viewing plane and defined rounded or flowing as a >90° angle of the osteophytes. Although both decisions concur with the original Resnick criteria they can be considered arbitrary.

In summary, the present study indicates that introducing modifications to the original Resnick criteria to diagnose DISH on CTs leads to moderate to excellent agreement between observers with different degrees of experience and expertise. In daily practice these modified Resnick criteria can be readily deployed in CT assessments of the thoracic spine. Further research validating this approach and correlating it to DISH-related outcomes is warranted.
